# XGBG: A Novel Method for Identifying Ovarian Carcinoma Susceptible Genes Based on Deep Learning

**DOI:** 10.3389/fonc.2022.897503

**Published:** 2022-05-12

**Authors:** Ke Feng Sun, Li Min Sun, Dong Zhou, Ying Ying Chen, Xi Wen Hao, Hong Ruo Liu, Xin Liu, Jing Jing Chen

**Affiliations:** ^1^ Department of Obstetrics and Gynecology, First Affiliated Hospital, Heilongjiang University of Chinese Medicine, Harbin, China; ^2^ Department of Oncology, The Second Affiliated Hospital of Dalian Medical University, Dalian, China; ^3^ Department of Oncology, Affiliated Zhongshan Hospital of Dalian University, Dalian, China; ^4^ Department of Nephrology, The First Affiliated Hospital of Heilongjiang University of Chinese Medical, Harbin, China; ^5^ Heilongjiang University of Chinese Medicine, Harbin, China; ^6^ Department of Rheumatology and Immunology, The First Hospital Affiliated to Army Medical University, Chongqing, China

**Keywords:** ovarian cancer, susceptible genes, XGBG, deep learning, pathway analyses

## Abstract

Ovarian carcinomas (OCs) represent a heterogeneous group of neoplasms consisting of several entities with pathogenesis, molecular profiles, multiple risk factors, and outcomes. OC has been regarded as the most lethal cancer among women all around the world. There are at least five main types of OCs classified by the fifth edition of the World Health Organization of tumors: high-/low-grade serous carcinoma, mucinous carcinoma, clear cell carcinoma, and endometrioid carcinoma. With the improved knowledge of genome-wide association study (GWAS) and expression quantitative trait locus (eQTL) analyses, the knowledge of genomic landscape of complex diseases has been uncovered in large measure. Moreover, pathway analyses also play an important role in exploring the underlying mechanism of complex diseases by providing curated pathway models and information about molecular dynamics and cellular processes. To investigate OCs deeper, we introduced a novel disease susceptible gene prediction method, XGBG, which could be used in identifying OC-related genes based on different omics data and deep learning methods. We first employed the graph convolutional network (GCN) to reconstruct the gene features based on both gene feature and network topological structure. Then, a boosting method is utilized to predict OC susceptible genes. As a result, our model achieved a high AUC of 0.7541 and an AUPR of 0.8051, which indicates the effectiveness of the XGPG. Based on the newly predicted OC susceptible genes, we gathered and researched related literatures to provide strong support to the results, which may help in understanding the pathogenesis and mechanisms of the disease.

## Introduction

Ovarian carcinomas (OCs) are one of the most fatal cancers in women; a scientific study of the disease is of vital priority due to its high death rate (1). A better understanding of the entities and molecules that contribute to the pathogenesis and progression of OC is essential to improve the diagnostics and treatment of the disease. Although the etiologic causes of OCs have not been recognized well, genetic factors that caused mutations in the disease have been examined profoundly with the help of many genetic approaches. However, there are still many disease susceptible genes not identified, and it is of vital importance to explore the mechanism and underlying pathogenic factors to better understand the disease and make a contribution in treating the disease.

A genome-wide association study (GWAS) is an approach utilized in genetics research to associate specific genetic variants [single-nucleotide polymorphisms (SNPs)] with a specific disease. It has identified hundreds of risk genetic variants (SNPs) that may result in ovarian cancers (2–6). However, these studies can only explain a small fraction of disease-related regions in a functional point of view (7–9). Since many risk alleles may locate in the non-protein-coding regions to regulate the expression of target genes (10), though GWAS provides strong support in revealing the associations between variants and traits, it is not comprehensive to discover the disease-related genes or gene regulators merely based on GWAS datasets.

Expression quantitative trait loci (eQTLs) are genomic loci that explain variation in expression levels of genes, which can be regarded as an additional evidence for identifying disease-related genes. eQTLs indicate the chromosomal loci that can explain variance in expression traits. These distinguishing characteristics from most expression quantitative trains are not the product of the expression of a single gene. With the help of eQTL analyses, a lot of causal genes for multiple types of cancers have been identified, such as kidney cancers, prostate cancers, breast cancers (9, 11, 12), and other complex diseases such as Alzheimer’s disease and schizophrenia (13, 14). Therefore, it is more worthy to discover disease causal genes based on the integration of both GWAS and eQTL datasets.

In addition to the genetic information derived from GWAS and eQTL datasets to understand the mechanisms of complex diseases, investigation and identification of molecular pathways are also important in exploring the underlying mechanism of diseases. Pathway analysis is a typical efficient analysis to explore the biology of genes and proteins that are differentially expressed in biological processes. There are many widely accepted pathway databases such as KEGG and BioCarta that can provide illustrative information to study diseases from the view of pathway system (15, 16). According to the information of molecular dynamics and cellular processes, genes and gene products are annotated based on different functions and characteristics (17). Since complex diseases are not only caused by a single gene or a single biological process, it is important to understand the diseases and identify disease causal genes from the point of view of a pathway system.

In this article, we proposed a novel OC causal gene identification method, XGPG, integrating gene features from both genomic point and pathway annotation point. We first employed the graph convolutional network (GCN) to reconstruct the gene feature based on both gene feature and network topological structure, then utilized a boosting method, extreme gradient boosting (XGBoost), to predict OC-related susceptible genes as a binary classification problem. By applying this method, we built an efficient gene prediction model and prioritized more putative genes associated with OCs.

## Methods

### Framework

**Figure 1:**
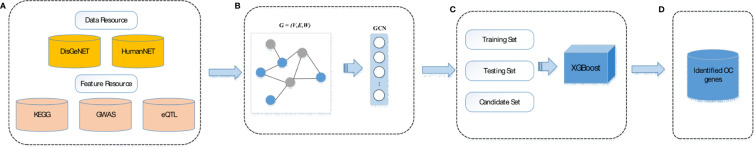
Work frame of the XGBG model. **(A)** Data resource; **(B)** GCN workflow; **(C)** XGboost workflow; **(D)** final classifier.

Our method, XGPG, contains 4 main parts, data collection, feature extraction, gene feature reconstruction based on both gene feature and network topology structure, and OC causal gene prediction based on the constructed XGBoost model. In the first section (A), we manually collected different types of ovarian diseases including OC-related genes from the DisGeNET database (18) and then we obtained gene features from the GWAS Catalog, GTEx Portal, and KEGG database for different features (19, 20). Furthermore, we collected gene interaction information from the HumanNet database (21). (B) Thus, we extracted gene features from GWAS data, eQTL data, and pathway annotations, and then extracted gene network structure topological features based on the gene–gene interaction network. (C) After the feature extraction process, we utilized the GCN model to reconstruct the integrated gene features based on both gene feature and topological structure for a more precise representation of collected genes. (D) In the disease gene prediction part, a boosting model, XGBoost, is employed for constructing the prediction model and to prioritize OC-related genes. The work frame is shown in **Figure 1**.

### Data Collection

We first downloaded published verified ovarian cancer-related genes from the DisGeNET database; after filtering, the dataset contains 3,181 genes to be regarded as a positive gene set. To construct a balanced training set, we randomly selected 3,171 genes that have interactions with positive genes but have no associations with ovarian diseases. These genes are used to construct the negative gene set. Then, we downloaded gene interaction information from the HumanNet database to build the gene–gene interaction network. For the prediction of OC causal genes, we also downloaded 721 ovarian disease-related genes as candidate genes to construct the prediction gene set. To extract gene features, we downloaded GWAS data from the GWAS Catalog and obtained 9,793,553 susceptible loci associated with OC, and we downloaded eQTL data from the GTEx v8 database including 25,325 susceptible loci detected in ovary tissue based on gene expression level. Moreover, we downloaded gene-pathway information from the KEGG database, including 343 annotated pathways.

### Feature Extraction

We extracted gene features from three aspects, namely, GWAS data, eQTL, data and KEGG pathway information. We first obtained the detailed gene location information of the training and predictive gene data, including chromosome name, start position, and end position. Then, the genes are mapped to the SNPs provided by GWAS data. To construct the SNP feature, we sorted the gene-mapped SNPs by *p*-value and extracted the top 5 significant SNPs as the SNP feature of the gene. Thus, the SNP feature can be denoted as a 5-D vector:


(1) $$F_{SNP}=\left[D_{1},D_{2},\;D_{3},D_{4},\;D_{5}\right]$$

For those genes that have less than 5 mapped SNPs, we set the value to 9 × 10^−6^ to avoid calculation error. For the expression feature, we mapped the genes to eQTL data based on gene location information and then extracted the top 5 significant eQTL *p*-values as expression feature. We also set the value to 9 × 10^−6^ for those genes mapped to less than 5 loci to avoid the calculation error. Thus, the expression feature can be denoted as a 5-D vector:


(2) $$F_{exp}=\left[D_{1},D_{2},\;D_{3},D_{4},\;D_{5}\right]$$

We then downloaded the KGML files from the KEGG database, representing the details for computational analysis and pathway relations in KEGG pathways. According to the KGML files, we can obtain the genes that participate in each KEGG annotated pathway. In total, the KEGG database has annotated 343 pathways; thus, the pathway feature of each gene can be denoted as a 343-D vector; the value is set to 1 if the gene is in the pathway process or set to 0 *vice versa*:


(3) $$F_{path}=\left[D_{1},D_{2},\ldots ,D_{342},D_{343}\right]$$

(4) $$D_{i}=\{0,\;\text{ if gene is in pathway }i1,\text{if gene is not in pathway}i$$

Thus, the primary feature representation of each gene can be denoted as a 353-D vector including the SNP feature, the expression feature, and the pathway feature. Since the feature matrix could be very sparse and is not comprehensive, we further utilized the GCN model to reconstruct the feature representation with the information of the gene interaction network topological structure.

### Feature Reconstruction by GCN

We first downloaded the gene–gene interaction information from the HumanNet database and constructed a gene–interaction network of the training set with dimensions of 6,352 × 6,352. Then, the adjacent matrix can be constructed based on the topological structure of the net. Next, the gene interaction network with gene features is input to the GCN model to reconstruct the gene features to obtain a more comprehensive feature representation. Consider the graph G = (V, E, W), where V is the nodes, E is the edge, and W is the weight matrix encoding the associations between nodes. In the GCN model, Rectified Linear Units (ReLU) is used as the activation function. We input the gene feature matrix X to the GCN model and then the gene feature can be extracted by the propagation rule of each layer:


(5) $$H^{l+1}=\;\sigma \left(LH^{l}XW^{l}\right)$$

(6) ReLU(x)= {x,x>0 0,x≤0 

where *σ* is the non-linearity activation function; here, we used ReLU.

Lastly, gene feature representation is reconstructed by GCN.

### Gene Prediction Based on XGBoost Model

XGBoost is a state-of-the-art boosting method that has been widely employed in many kinds of data mining problems. It can also be used in classification and regression problems. Boosting is an ensemble learning algorithm that firstly train a weak model and then train an enhanced model to improve the errors by iteration. By iteration, the new model can fit the residuals of the previous model. Here, we utilized the “xgboost” package in R to perform the training and prediction process. In order to evaluate our prediction model, we performed a 10-fold cross-validation on the 6,352 training set. Since the training set is composed of 3,181 positive samples and 3,171 negative samples, we randomly divided them into 10 groups, and 9 of them is used to train the model and the last one is used to test the model based on the labels at each time. Grid searches were performed to evaluate the best performance of the parameters of the model.

## Results

### Measurement of Model Performance

**Figure 2:**
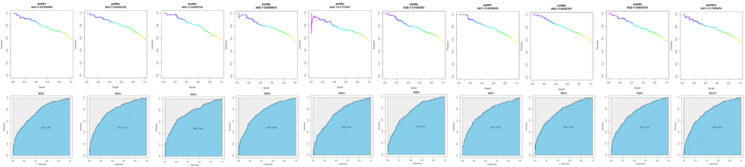
Ten CV performance of the XGPG model.

Since we have assessed the performance of our model based on 10 CVs with training sets, the ROC curve and PR curve are used to measure the performance of the model; the curves of 10 CVs are shown in **Figure 2**. The AUC and AUPR of 10 CVs are shown in **Table 1**. As a result, we obtained the average AUPR of 0.8051 and the average AUC of 0.7541. We chose the best performance model with an AUPR of 0.8301 and an AUC of 0.7770 to predict the OC causal genes.

**Table 1:** AUPR and AUC of 10 CVs.

	1	2	3	4	5	6	7	8	9	10	Ave
AUPR	0.8102	0.8301	0.8187	0.8050	0.7731	0.7959	0.8034	0.8191	0.8050	0.7904	0.8051
AUC	0.7484	0.7770	0.7558	0.7592	0.7294	0.7568	0.7533	0.7564	0.7516	0.7532	0.7541

### Performance Comparison Between Models

**Figure 3:**
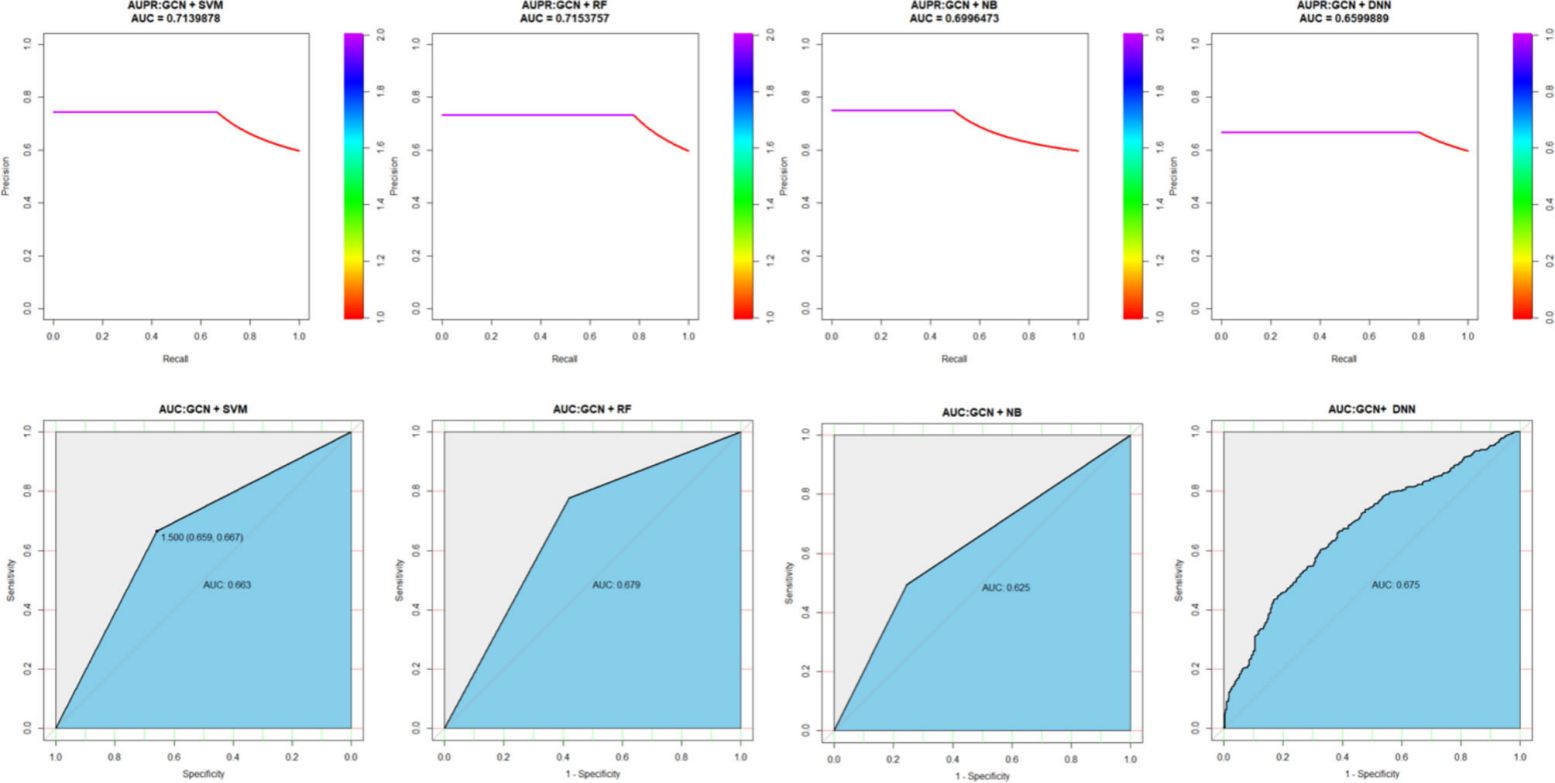
Performance comparison with different models.

Although we have proved the performance of XGPG by 10 CVs on the training set, there have been many other machine learning and deep learning methods used in classification problems, such as random forest (RF), Naïve Bayesian (NB), support vector machine (SVM), and deep neural network (DNN). To better illustrate the effectiveness and credibility of XGPG, we also compared it with SVM, RF, Naïve Bayes, and DNN. In order to ensure the consensus of the input to each model, all the gene features are reconstructed by GCN. The results are shown in **Figure 3**. As shown in the figure, SVM and RF perform better than NB and DNN, but they are far behind the XGBoost model.

### OC Gene Prediction Process

Since we have demonstrated the performance of our method and chose the best model to predict the OC genes, we then performed the gene prediction process with 721 verified ovary disease-related genes obtained from DisGeNET to further identify genes that are significantly associated with OCs. We also extracted the gene features as mentioned in the *Feature Extraction* section and built the gene interaction network to obtain the topological structure. After the gene prediction process by XGPG, we finally prioritize the candidate genes by the score resulting from the XGBoost model.

## Case Study

According to the results, our method predicted 148 (score threshold is 0.8) and 45 (score threshold is 0.9) OC causal genes from 721 candidate susceptible genes. We listed the top 20 genes in **Table 2**. As shown in **Table 2**, some of predicted genes have been reported to have direct or indirect associations with OC. Studies have indicated that KNG1 is highly related to the gonadotropin-releasing hormone (GnRH) (22), which is a hypothalamic neuropeptide that plays an important role in the reproductive system. Investigators have made a great effort to develop GnRH agonists and antagonists for the treatment of tumors such as ovarian cancers (23). Coagulation factor II (F2) is found to be overexpressed in various epithelial neoplasms including ovarian cancer (24); F2 receptor, also known as PAR1, has been provided to be differentially expressed in ovarian cancer tissue (25). F13A, also known as coagulation factor XIII A, has been proven to have a significantly higher concentration in OC plasma, which may be a powerful tool for the clinical diagnosis and prognostic prediction of the disease (26). RASA1 is a member of the RAS-GAP family, which has been reported to play an important role in cell proliferation and migration in several types of cancers, including OC, by inhibiting the malignant progression of OC cells in a high level (27). Furthermore, SMAD1 can regulate BMPs (such as BMPR1A), resulting in aberrant BMP signaling in ovarian cancer pathology (28, 29). The IGF system has been implicated in OC since it has a key role in normal growth and development. In the Yang study, they proved that IGFBP-6 may have profound effects on the migration of two ovarian cancer cell lines, which may help in developing an IGFBP-6-based therapeutic for ovarian cancers (30). Since AGTR1 has been demonstrated to be the main effector of RAS and AGTR1 protein was detected in 86% of OC tissues, AGTR2 is the antagonist of AGTR1, which means that it also plays an important role in the pathology of OC (31). NPPB is a secreted protein that has been proven to maintain a high level in the blood of women with ovarian cancer, which indicates that NPPB may be a novel biomarker for the detection of EOC (32).

**Table 2:** Top 20 predicted OC causal genes.

Symbol	NCBI ID	Symbol	NCBI ID
3827	KNG1	2147	F2
2162	F13A1	5577	PRKAR2B
5921	RASA1	4086	SMAD1
657	BMPR1A	58	ACTA1
2688	GH1	2690	GHR
1489	CTF1	3489	IGFBP6
186	AGTR2	4879	NPPB
22806	IKZF3	10370	CITED2
8204	NRIP1	406954	MIR181A2
407021	MIR29A	407036	MIR32

## Discussion

OCs are one of the most dangerous cancers for women. It is important and essential to understand the mechanisms of the disease. In this study, we proposed an OC causal gene prediction method, XGPG, based on the deep learning method and the boosting method. Since GWASs have identified lots of susceptible loci associated with OC, due to the theory of linkage disequilibrium (LD), SNPs can regulate the pathologies of traits on the expression level of target genes. Thus, we integrated both GWAS and eQTL data to integrate the gene feature from both genetic and expression levels. Moreover, since complex diseases are not only caused by a single gene or SNP, it is important to also take gene–gene interaction into consideration. We built the gene interaction network to extract the gene network topological structure. Based on both gene feature and structure feature, we can reconstruct the gene feature representation by the GCN model and then perform the prediction process using the XGBoost model. We obtained a high AUPR 0.8051 of and an AUC of 0.7541 on the training set composed of 3,181 positive samples and 3,171 negative samples after 10-fold cross-validation. Compared with 4 other models, SVM, RF, NB and DNN, our model performed much better. Then, we performed the OC prediction process on the 721 candidate genes and derived a prioritized gene list. As a result, our method predicted 148 (score threshold is 0.8) and 45 (score threshold is 0.9) OC causal genes. From the results, prioritized genes such as F13A, RASA, SMAD, and AGTR2, and several other genes are published and proved to be associated with OC, which also proved the effectiveness of our method. In summary, our method is helpful in further understanding the etiology and pathology of OC, and may be used as a strong theoretical evidence for drug design.

## Data Availability Statement

The original contributions presented in the study are included in the article/supplementary material. Further inquiries can be directed to the corresponding authors.

## Author Contributions

KS, LS, and DZ designed the experiments, analyzed the data, and wrote the manuscript. YC and XH analyzed the bioinformatic data. HL and XL provided important ideas. This whole work is guided by JC. All authors contributed to the article and approved the submitted version.

## Conflict of Interest

The authors declare that the research was conducted in the absence of any commercial or financial relationships that could be construed as a potential conflict of interest.

## Publisher’s Note

All claims expressed in this article are solely those of the authors and do not necessarily represent those of their affiliated organizations, or those of the publisher, the editors and the reviewers. Any product that may be evaluated in this article, or claim that may be made by its manufacturer, is not guaranteed or endorsed by the publisher.
